# Nrf2 and Heme Oxygenase-1 Involvement in Atherosclerosis Related Oxidative Stress

**DOI:** 10.3390/antiox10091463

**Published:** 2021-09-14

**Authors:** Jose Angel Alonso-Piñeiro, Almudena Gonzalez-Rovira, Ismael Sánchez-Gomar, Juan Antonio Moreno, Ma Carmen Durán-Ruiz

**Affiliations:** 1 Biomedicine, Biotechnology and Public Health Department, Cádiz University, 11519 Puerto Real, Spain; joseangel.alonsopi@alum.uca.es (J.A.A.-P.); almudena.gonzalez@uca.es (A.G.-R.); ismael.sanchez@uca.es (I.S.-G.); 2 Institute of Research and Innovation in Biomedical Sciences of Cádiz (INiBICA), 11001 Cádiz, Spain; 3 Maimonides Biomedical Research Institute of Cordoba (IMIBIC), UGC Nephrology, Hospital Universitario Reina Sofia, 14004 Cordoba, Spain; jamoreno@fjd.es; 4 Department of Cell Biology, Physiology, and Immunology, Agrifood Campus of International Excellence (ceiA3), University of Cordoba, 14014 Cordoba, Spain

**Keywords:** atherosclerosis, oxidative stress, heme oxygenase-1, Nrf2

## Abstract

Atherosclerosis remains the underlying process responsible for cardiovascular diseases and the high mortality rates associated. This chronic inflammatory disease progresses with the formation of occlusive atherosclerotic plaques over the inner walls of vascular vessels, with oxidative stress being an important element of this pathology. Oxidation of low-density lipoproteins (ox-LDL) induces endothelial dysfunction, foam cell activation, and inflammatory response, resulting in the formation of fatty streaks in the atherosclerotic wall. With this in mind, different approaches aim to reduce oxidative damage as a strategy to tackle the progression of atherosclerosis. Special attention has been paid in recent years to the transcription factor Nrf2 and its downstream-regulated protein heme oxygenase-1 (HO-1), both known to provide protection against atherosclerotic injury. In the current review, we summarize the involvement of oxidative stress in atherosclerosis, focusing on the role that these antioxidant molecules exert, as well as the potential therapeutic strategies applied to enhance their antioxidant and antiatherogenic properties.

## 1. Introduction

Atherosclerosis constitutes a chronic inflammatory disease affecting the middle and large arteries, which mainly initiates by infiltration and deposition of low-density lipoproteins (LDL) into the arterial intima, where they undergo oxidation [1]. This, in turn, promotes an inflammatory response which leads to leukocyte (monocytes mainly) mobilization and recruitment, uptake of oxidized-LDL (ox-LDL) particles and further deposition into the vascular walls, resulting in the formation of complex structures known as atherosclerotic plaques [2]. Although these plaques might remain stable, they usually progress, narrowing the vessel lumen diameters and thus limiting blood flow through the arteries. In the worst scenario, plaque rupture promotes the release of pro-thrombotic factors into the circulation, triggering the blockage of luminal vessels which will be responsible for ischemic strokes or myocardial infarction, among other such events [2,3,4]. 

Oxidative stress mediated by reactive oxygen species (ROS) plays a key role in the atherosclerotic process. ROS not only oxidize LDL, they also promote several pro-atherogenic effects, including inflammation, apoptosis, and alteration of vascular tone [5,6]. The imbalance between pro- and antioxidant agents drives to the formation and progression of atherosclerotic plaque lesions. In this sense, the transcription factor Nuclear factor–erythroid 2-related factor 2 (Nrf2) is known to activate the expression of more than 250 antioxidant enzymes—such as heme oxygenase-1 (HO-1), glutathione peroxidase, or glutamate-cysteine ligase [7,8]—making it a master regulator of oxidative stress. Nrf2 has been associated with different cardiovascular-related pathologies—such as obesity, diabetes mellitus, atherosclerosis, hypertension, or heart failure [9,10]. Currently, Nrf2 signaling pathway is considered an important defense mechanism against cardiovascular diseases (CVDs). On the other hand, Nrf2 appears to display contradictory effects in cancer [11], since its overexpression protects cells against diverse carcinogen agents [12], while aberrant expression of Nrf2 contributes to development and progression of several types of cancers [13]. Similarly, different studies have focused on the involvement of Nrf2 in neuroinflammatory diseases, such as Parkinson’s [14] or Alzheimer´s disease [15], where this factor may play a protective role, mainly by promoting reduction of oxidative damage and neuroinflammation [14]. Herein, the role of Nrf2 and HO-1 as potential targets against atherosclerosis-related oxidative stress has been reviewed.

## 2. Atherosclerosis

Atherosclerosis mainly affects two of the three artery layers: the endothelial and the middle layer. The endothelium is composed mainly of endothelial cells (ECs), representing the innermost layer of the artery. The endothelium constitutes a selectively permeable barrier responsible for maintaining arterial homeostasis by controlling the exchange of substances and the release of vasoconstrictor/vasodilator factors. This layer also regulates vascular tone, cell adhesion, and smooth muscle cells (SMCs) proliferation [16,17]. The tunica media constitutes the intermediate layer, which is separated from the previous one by an internal elastic lamina, and it contains mainly vascular smooth muscle cells (VSMCs). These cells control the basal tone through vasoconstriction and dilation of vessels, thus regulating blood pressure and ensuring appropriate blood flow. The third and final layer, composed mainly of fibroblasts, is the so-called adventitia [17,18]. 

### 2.1. Atherosclerosis Initiation

**Figure 1:**
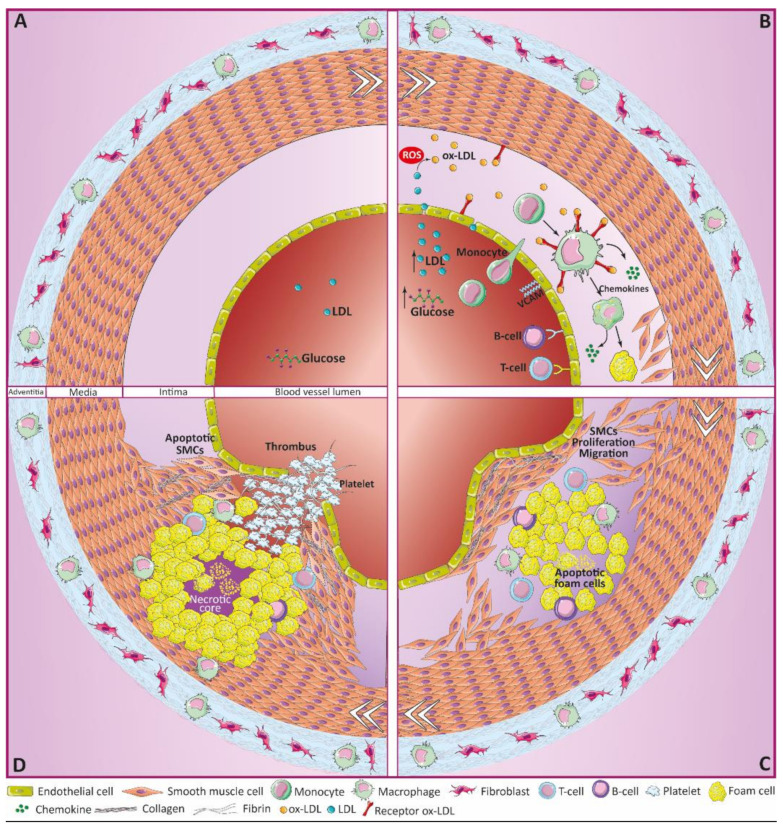
Atherosclerosis progression. (**A**) Different factors promote the initiation of atherosclerosis, including hyperglycemia or oxidative stress. (**B**) ox-LDL phagocytosis by monocyte/macrophages derives in accumulation and deposit of foam cells, and the recruitment of B and T lymphocytes. (**C**) Formation of fatty streaks, as well as proliferation and migration of SMCs towards the injured area, generate complex structures known as atherosclerotic plaques. Atherosclerotic plaques partially block the internal lumen of vascular vessels, reducing blood flow and oxygen/nutrient supply to surrounding tissues. (**D**) Plaque rupture activates thrombotic events, fully blocking the circulation, which might result in brain stroke or myocardial infarctions.

Under pathological conditions, many of the biological functions regulated by the endothelium are compromised, and such endothelial dysfunction constitutes the first step in the development of atherosclerosis (Figure 1A). Overall, the integrity of the endothelial layer can be affected by two types of factors [18]: physical factors (such as shear stress), or chemical agents like ox-LDL or high glucose levels. Endothelial dysfunction and apoptosis take place in arterial branches, where normal blood flow changes from laminar or high shear stress (HSS) to turbulent or low shear stress (LSS). Indeed, while laminar blood flow appears to protect against atherosclerosis, disturbed flow becomes pro-atherogenic [16,17]. For instance, under HSS flow, ECs synthesize nitric oxide (NO) by endothelial NO synthetase (eNOS), which acts as a vasodilator and inhibits apoptosis, neutrophil adhesion, macrophage chemoattractant cytokine production, as well as platelet aggregation [16]. On the other hand, disturbed flow enhances monocyte chemotactic protein-1 (MCP-1) expression and monocyte infiltration in ECs [19], downregulation of cell proliferation [20], and activation of EC apoptosis by enhancing mitochondrial cytochrome C release [16]. Similarly, shear stress upregulates the expression of vascular endothelial growth factor (VEGF), which potentiates endothelial permeability [21]. Moreover, LSS is known to stimulate ROS production by ECs, which potentiates, among others, the expression of intercellular adhesion molecule-1 (ICAM-1) and subsequent leukocyte adhesion [22].

ECs are also susceptible to high concentrations of different compounds, including ox-LDL or high glucose levels, among others (Figure 1B). LDLs are well known lipid storing molecules [23] whose oxidation becomes toxic to the endothelium, promoting apoptosis in human umbilical vein endothelial cells (HUVECs) and human aortic endothelial cells (HAECs) [24]. Indeed, reduction of ox-LDL levels by simvastatin seems to decrease apoptosis in HUVECs [25]. Remarkably, ox-LDL tends to accumulate in areas in which arteries are exposed to turbulent flows and LSS [26]. Hyperglycemia also induces ECs dysfunction, over-production of ROS [27,28] and NO synthesis blockade [16], becoming very toxic for HUVECS [27,28]. Finally, overproduction of ROS—induced by high-glucose levels, LSS, or other factors—promotes ECs apoptosis, increased oxidation of LDL particles and endothelial dysfunction. Overall, all these factors compromise the integrity of the endothelial layer and the endothelial extracellular matrix (ECM). As result, increased permeability of ECs facilitates the infiltration of inflammatory cells and even more LDL into the arterial wall [16,29], representing the initiation of the atherosclerotic process.

### 2.2. Early Stages of Atherosclerosis

LDL molecules are recognized in the middle layer by scavengers receptors (SR) on the cell-surface of macrophages and SMCs, whose expression is also induced by ROS [30] (Figure 1B). ox-LDL uptake stimulates the activation of lysosomal acid lipase, which releases cholesterol from LDL by hydrolyzing cholesterol esters. When the levels of phagocyted ox-LDL are too high, the lipase activity is inhibited and cholesterol ester droplets start to accumulate inside the lysosome. Besides, part of this cholesterol leaves the lysosome and can be re-esterified, forming lipid clusters into the cytosol. Lipid deposition reduce the ability of cells to migrate, becoming trapped inside the arterial wall, and turning into foam cells [16,23,26]. Although most foam cells derive from infiltrated macrophages, a small part come from ECs and VSMCs [26]. Accumulation of foam cells in the arterial middle layers results in structures known as fatty streaks, which act as precursors of major lesions [26,31].

Lipids and foam cells not only act as initiators of the atherosclerotic process, they also maintain inflammation, and aggravate it. For instance, lipids bind to toll-like receptors, which activates the synthesis of inflammatory chemokines, promoting the recruitment of additional macrophages together with B and T cells towards the injured area [16,23]. Moreover, in response to lipid deposition, ECs synthesize different cytokines and adhesion molecules such as vascular cell adhesion molecule 1 (VCAM-1), which also recruit inflammatory cells, causing monocytes differentiation into macrophages. These cytokines enhance the production of proteins, such as ox-LDL receptor-1 (LOX-1) in ECs, which makes them more permeable to ox-LDL [26].

### 2.3. Atheroma Plaque Progression

The deposition of lipid-rich cell debris within the damaged area takes place as result of apoptosis of foam cells and immune cells, building-up the “necrotic core” [26,29]. VSMCs will then migrate to the necrotic core, where they accumulate and secrete ECM proteins, forming a fibrous layer which stabilizes the atherosclerotic plaque [16,29] (Figure 1C). At this point, several factors support plaque stabilization, while others disrupt it, ending in plaque rupture. For example, stable atherosclerotic plaques are rich in phospholipids and triglycerides, while unstable ones contain high levels of cholesterol and free cholesterol esters [23]. Besides, atherosclerotic plaques with larger fibrous layers are more stable and less prone to rupture [18]. Usually, vulnerable plaques correlate with higher proteolytic activity and inflammation within the atherosclerotic area [32]. Proteolytic enzymes degrade the fibrous cap, increasing the probability of plaque rupture and atherothrombotic events [33], which are aggravated by the accumulation of platelets within the area [34] (Figure 1D).

## 3. Atherosclerosis and Oxidative Stress

**Figure 2:**
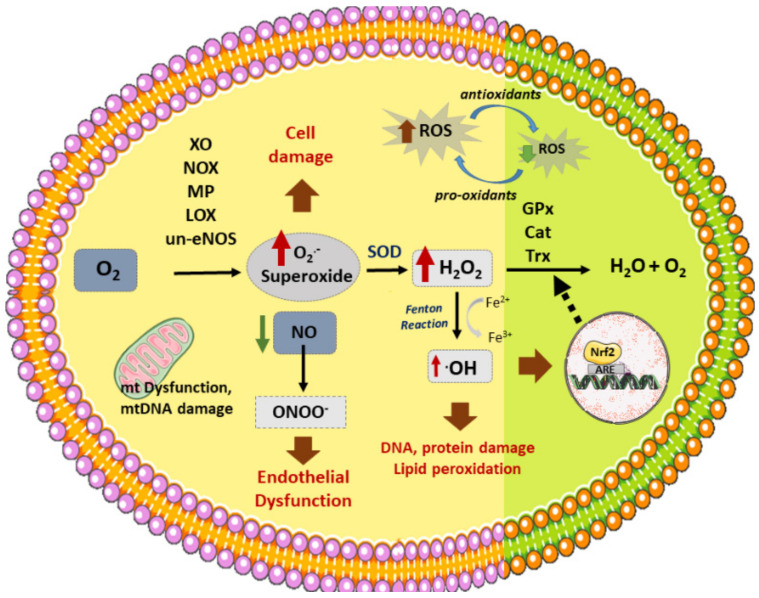
Vascular sources of ROS and related enzymes. Different enzymes participate in the formation of ROS: NOX (NADPH oxidase), XO (xanthine oxidase), un-eNOS (uncoupled nitric oxide synthase), LOX (lipoxygenase), MP (myeloperoxidase), generating superoxide (O_2_^.-^) from O_2_. In addition, dysfunctional mitochondrial (mt) respiratory chain is another source of (O_2_^.₋^). On the other hand, SOD (superoxide dismutase) produces H_2_O_2_ (hydrogen peroxide) from superoxide, which can then be converted to H_2_O by several antioxidant enzymes: GPx (glutathione peroxidase), Cat (catalase), or Trx (Thyoredoxin). H_2_O_2_ reacts with transition metals such as Fe^2+^ (through the Fenton reaction) to produce hydroxyl radicals (^.^OH). Nitric oxide (NO) reacts with O_2_^.₋^ to produce peroxynitrite (ONOO₋). ROS stimulates Nrf2 activation and translocation to the nucleus, activating the synthesis of antioxidant enzymes. ROS production induces cell death, DNA and lipid peroxidation, and endothelial dysfunction, among other effects, which triggers the atherosclerotic process.

As described above, oxidative stress is highly involved in the initiation and development of atherosclerosis [30]. Apart from the active oxygen compounds, free radicals (O_2_**^.₋^**, **^.^**OH) and non-free radicals (H_2_O_2_, ½ O_2_), reactive nitrogen, copper, iron and sulfur species are also considered as oxidative stress related molecules, since they promote or participate through different mechanisms in the synthesis of ROS [5,30]. ROS production increases in response to many risk factors associated with atherosclerotic CVDs—such as hypertension, diabetes, smoking, or dyslipidemia [30]. Similarly, oxidative stress can result from the imbalance of oxidant and antioxidant agents, promoting cell toxicity related to atherosclerosis [17]. Under physiological conditions, antioxidants help to prevent damage from free radicals and other oxidant molecules without affecting redox reactions that are fundamental for appropriate cell function [35]. However, under pathological situations, an imbalance of oxidant (enhanced) versus antioxidant enzymes (reduced) leads to oxidative stress (Figure 2). Then, many different processes can take place, including NO inactivation; oxidation of lipids, proteins, or even DNA; cell apoptosis; or enhanced expression of pro-inflammatory cytokines and ox-LDL receptors (CD36, LOX-1) [36,37]. Remarkably, ROS promotes the expression of SR in VSMCs, ox-LDL recognition and uptake, as well as their transformation into foam cells [30]. In the same way, ROS can activate the release of matrix metalloproteinases (MMPs) which could degrade the basement membrane and atheroma plaque structure, disrupting it. Additionally, ECs and SMCs can generate different oxidant agents through several enzymes [38].

### 3.1. Vascular Sources of ROS

At the vascular level, several endogenous enzymes are known to participate as major sources of ROS, including nicotinamide adenine dinucleotide phosphate (NADPH) oxidase (NOX), xanthine oxidase (XO), lipoxygenase, myeloperoxidases (MP), or uncoupled endothelial NO synthase (eNOS) (Figure 2) [39].

The NOX family is comprised of seven isoforms of NADPH oxidases, all transmembrane proteins with multiple cytosol and cell membrane subunits (reviewed in [40,41]). These enzymes reduce O_2_ to superoxide (O_2_**^.₋^**) by transferring one electron from NADH or NADPH to O_2_. Superoxide radicals react with NO producing peroxynitrite (ONOO₋), a cytotoxic molecule that contributes to EC injury. This reaction decreases NO bioavailability, together with its vasorelaxant effect [42]. NADPH oxidases are stimulated by pre-atherosclerotic inducers such as shear stress, angiotensin II, or growth factors such as TGFβ or PDGF [42]. Thus, while isoforms Nox1 and Nox2 have been shown to promote pro-atherosclerotic effects [39,43], the involvement of Nox4 in the atherosclerotic process seems more complex. Nox4 appears upregulated in ApoE null mice (ApoE^₋/₋^) [44] and elevated levels of this enzyme in human arteries have been linked to the severity of atherosclerosis [45]. On the other hand, Nox4 atheroprotective effects have also been described in diabetic atherosclerotic mice, by modulation of VSMC proliferation [46].

NOX enzymes also promote the oxidation of tetrahydrobiopterin (BH4), the main cofactor of eNOS. As a result, eNOS, principal producer of endothelial NO during phy-siological conditions, becomes dysfunctional, reducing the levels of this vasodilator and antiatherogenic agent [42]. Moreover, eNOS uncoupling from the NO synthesis results in the production of O_2_**^.₋^**, ONOO^₋^, and H_2_O_2_, therefore increasing oxidative stress products, which increases endothelial dysfunction and atherosclerosis initiation [47].

XO catalyzes the oxidation of hypoxanthine to xanthine and xanthine to uric acid. In this reaction, XO uses O_2_ as electron donor, producing NADH, H_2_O_2_, and O_2_**^.₋^**. Different stimuli can activate the expression of XO, including shear stress, hypoxia, or proinflammatory factors such as IL-6 or TNF-α [42]. XO has been associated with different CVDs [48]. For instance, upregulation of XO has been found in patients with coronary artery disease [49], while XO and uric acid were found in high concentrations in carotid atherosclerotic plaques, colocalizing with cholesterol in SMCs and ECs [50]. 

Finally, mitochondrial dysfunction can also drive to excessive ROS formation, as well as mitochondrial DNA (mtDNA) damage. Remarkably, mtDNA damage promotes itself ROS production. Both processes have been found associated with the development of a-therosclerosis [51,52]. For instance, mtDNA damage correlates with increased atherosclerotic occlusion of coronary arteries [53], as well as with the extent of atherosclerosis lesions in ApoE knockout mice [54]. 

### 3.2. Vascular Antioxidant Enzymes

Different antioxidant systems—such as glutathione peroxidase (GPx), superoxide dismutase (SOD), catalase (Cat), or thioredoxins (Trx)—can be found within the vascular wall. All these enzymes play an important role in neutralizing ROS production and reducing oxidative stress [55]. In this process, one of the major regulators of defense against ROS is the Nrf2 signaling pathway. Nrf2 not only upregulates the expression of different antioxidant enzymes, Nrf2 also modulates mitochondrial ROS production as well as NADPH oxidase activity [56].

## 4. NRF2 Antioxidant Roles in Atherosclerosis

Nrf2, encoded by NFE2L2 gene, constitutes a transcription factor that has been closely linked to atherosclerosis, although its specific role in this pathology is not clear. Nrf2 might play antagonistic roles, both preventing and enhancing atherosclerotic development [57,58,59]. For instance, several studies related to laminar and oscillatory blood flow have revealed that the former stimulates the antiatherogenic activation of Nrf2, while the latter promotes the opposite effect [60]. Studies with ApoE^₋/₋^ and Nrf2^₋/₋^ or Nrf2^+/₋^ mice indicate a reduction of the atherosclerotic process in these animals, suggesting a deleterious role of the transcription factor [57,61]. Nrf2 expression in ApoE Knockout mice seems to accelerate the late stages—but not the early stages—of atherosclerosis [62], while in absence of macrophage Nrf2, both atherosclerotic stages are affected in LDLR KO mice [58]. Nrf2 might potentiate atherosclerosis through different pathways, as described [63], promoting among others the formation of foam cells [64] or the monocyte recruitment to the lesion areas by upregulation of IL-1α in macrophages [57]. Nrf2 modulates the expression of CD36 scavenger receptor in macrophages, responsible for ox-LDL uptake and accumulation and subsequent formation of foam cells [65]. In this regard, controversial results have been assigned to the Nrf2/CD36 pathway in atherosclerosis, with studies indicating reduced ox-LDL accumulation in Nrf2 KO macrophages and decreased CD36 levels in atherosclerotic lesions in ApoE^₋/₋^ Nrf2^₋/₋^ mice [62,65]. Conversely, lack of Nrf2 in peritoneal macrophages seems to increase modified LDL uptake, suggesting that CD36 is not the only factor responsible for ox-LDL accumulation [58,65]. 

The majority of studies, however, have focused on the athero-protective properties of Nrf2, mainly as a key agent against oxidative stress [10]. Apart from being able to activate the expression of many antioxidant enzymes, Nrf2 has the potential to modulate NADPH oxidase activity as well. Thus, the levels of pro-atherogenic Nox2 appear to increase significantly in absence of Nrf2, whereas Nox4 is upregulated when Nrf2 is constitutively activated [56,66]. Interestingly, ROS produced by NADPH oxidase can also activate Nrf2, at least in cardiomyocites and pulmonary epithelial cells [67]. Such activation may represent an endogenous protecting mechanism against mitochondrial damage and cell death in the heart during chronic pressure overload [68]. Studies with Nrf2-KO mouse glio-neuronal cells have shown higher rates of mitochondrial ROS production in these cells than in wild type Nrf2 cells, suggesting a modulatory role for Nrf2 over the mitochondrial respiratory chain [56]. Indeed, Nrf2 enhances mitochondrial biogenesis, as well as the activation of OxPhos, among others. Finally, mitochondrial ROS also stimulates Nrf2 activation, thus representing a continuous regulatory loop [56,69].

Nrf2 not only protects against oxidative stress in ECs through activation of different antioxidant genes, it also exerts anti-inflammatory and angiogenic effects on these cells [70]. Indeed, Nrf2 suppresses the expression of VCAM-1 and TNFα—induced expression of MCP-1 in HUVECs [71] and HAECs [72]. Likewise, endothelial Nrf2 antioxidant response can be activated in areas exposed to oscillatory shear stress, exerting an athero-protective role [73]. Similarly, Nrf2 reduces the inflammatory response in macrophages and foam cells by modulation of several inflammatory cytokines such as IL-6 or TNFα [58]. Finally, Nrf2 could protect VSMCs against oxidative stress, and may protect against atherosclerosis progression by activation of athero-protective genes, such as HO-1 or NAD(P)H dehydrogenase quinone 1 (NQO1), which suppress proliferation of SMCs and VSMCs respectively [74,75].

### Modulation of Nrf2 Activity

**Figure 3:**
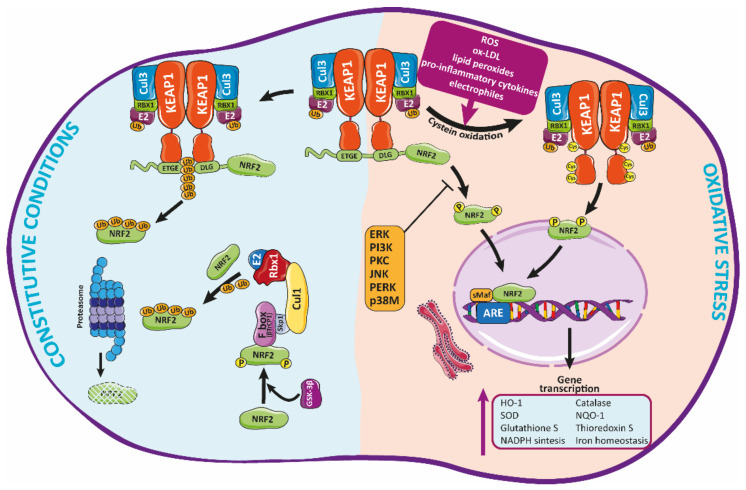
Regulation of Nrf2 signaling and HO-1 expression. Under basal conditions, Nrf2 is trapped into the cytosol associated with the Kelch-like ECH-associated protein-1 (KEAP-1) through the N-terminal (Neh2 domain). Herein, Nrf2 is transferred to proteasomal degradation after being ubi-quinylated. In response to pathological agents such as ROS, Nrf2 is released from this complex after oxidation of Keap1 cysteine residues, moving toward the nucleus, where it binds to ARE regions, inducing the expression of antioxidant enzymes such as HO-1.

Nrf2 belongs to “Cap’n’Collar” (CNC) family of transcription factors with a b-ZIP domain [76,77] which modulate the cellular redox status [76]. Nrf2 has seven domains that regulate the stability and functional activity of this factor [78]. Under normal homeostatic conditions, Nrf2 is trapped in the cytosol, associated with the Kelch-like ECH-associated protein-1 (KEAP-1) through the N-terminal (Neh2 domain) [78] (Figure 3). Keap1 acts as an endogenous inhibitor of Nrf2 [79]. In fact, Keap1 is the major regulator of Nrf2 activity [80], through the aforementioned interaction with Nrf2, which promotes the assembling with the Cullin3 (Cul3)/Rbx1 (Ring box-1)-based E3-ubiquitin ligase complex that targets Nrf2 for constant proteasomal degradation [78]. This complex keeps Nrf2 in the cytosol, avoiding its translocation to the nucleus and the transcription of antioxidant related genes [80].

Many factors—such as ROS, ox-LDL, lipid peroxides and their metabolites, electrophiles, pro-inflammatory cytokines, and many other molecules related to oxidative stress—can induce an alteration of Keap1 conformation (by modification of its cysteines residues) [7,64], promoting the release of Nrf2 which then translocates to the nucleus [64]. Herein, Nrf2 binds through the Neh1 functional domain to small musculoaponeurotic fibrosarcoma (sMaf) proteins, forming heterodimers with them [8]. Such dimerization allows Nrf2 to interact with the DNA through the antioxidant response element (ARE) sequence in specific gene regulatory regions [78]. Through this mechanism, Nrf2 modulates the expression of many genes encoding different antioxidant proteins—e.g., HO-1, catalase, SOD, or NQO-1 [81] among others—which will contribute to reduce cellular oxidative stress [64,82]. Likewise, Nrf2 regulates the glutathione and thioredoxin systems, NADPH production and utilization, and iron homeostasis. Many of these antioxidant factors actively participate in the detoxification and elimination of pro-oxidant compounds [81]. 

Currently, the Nrf2/Keap1 system is considered the principal mechanism against o-xidative stress, which is widely conserved, mainly in mammals [83]. This system is mainly modulated, as indicated above, by the interaction of diverse substances with several cysteines residues present in Keap1. Indeed, Keap1 contains more than 27 cysteine residues that have been described as potentially involved in the modulation of this protein and its interaction with Nrf2 [84]. Among them, Cys151 promotes Nrf2 release and activation, while Cys273, 288, and 297 facilitate the interaction of Keap1 with Nrf2, therefore inactivating this protein [85]. The current model postulates that, in response to different stimuli, modification of Keap1 thiols promotes conformational changes that negatively affect the ubiquitin ligase activity of the Keap1-Cul3 complex, as described before, releasing Nrf2 which then translocates to the nucleus and activates the synthesis of antioxidant genes [80]. Alternatively, the “hinge and latch” model suggests that, in response to stimuli, Nrf2 is synthesized de novo, instead of being released from Keap1, accumulating as well in the nucleus [86]. 

The Nrf2/Keap1 system can also be regulated by the autophagy regulator protein p62 (Sequestosome1, SQSTM1). Phosphorylation of p62 in response to many factors such as oxidative stress [87] allows the direct binging of this protein to Keap1, promoting its degradation by autophagia, and subsequent displacement and activation of Nrf2 [88]. Remarkably, p62 expression is also induced by Nrf2 in response to ROS [89]. Thus, the p62/Nrf2 route might represent an attractive candidate to promote Nrf2 activation and antioxidant response. However, given the strong correlation found between p62 upregulation and activation of Nrf2 pathway with several cancer types, major efforts have been made mainly toward the identification of potential pharmacological inhibitors of this p62/Nrf2 pathway [90].

Apart from Keap1, other molecules can regulate Nrf2 activity through additional control points at transcriptional, post-transcriptional, translational, and post-translational level [7,91] (Table 1). Several kinases, such as glycogen synthase kinase-3-beta (GSK3β), have been described as modulators of Nrf2 activity [92]. Nrf2 phosphorylation by GSK-3β activates the recognition and binding of Nrf2 to β-transducin repeat-containing protein (β-TrCP), an adaptor for the Skp1-Cul1-Rbx1-F-box protein (SCF) E3 ubiquitin ligase complex. Similarly to the Nrf2-Keap1-Cul3 complex, this complex promotes Nrf2 ubiquitination and further proteasomal degradation [93,94]. Other kinases—such as protein kinase-C (PKC), extracellular signal-regulated kinase (ERK), PERK, p38 MAPK, JNK, and PI3K—can also influence Nrf2 activity [59,95,96]. Remarkably, while phosphorylation of certain residues of Nrf2 (Ser40, Ser558) by kinases like PKC or AMPK-mediated phosphorylation enhance nuclear translocation of Nrf2 and transcription of antioxidant enzymes [95,96], phosphorylation or other residues (Tyr576, Ser44, Ser347) by enzymes such as Fyn kinase or GSK3B, can promote Nrf2 to be exported from the nucleus to the cytosol, which activates its proteasomal degradation [93]. 

Alternatively, several studies suggest that different microRNAs (miRNAs) (i.e., miR-27a, miRNA-28a or -34a) can also regulate, directly or indirectly, Nrf2 expression and moreover, to control the antioxidant activity of the Keap1-Nrf2 complex and Nrf2/ARE signaling pathways [91,112]. miRNAs are small noncoding molecules that bind to specific regions of mRNA (UTRs), promoting either their degradation or blocking their translation, reducing their levels. Thus, several miRNAs—such as miR-28, miR-507, mi-450a or miR-634—have been found to inhibit Nrf2 expression in studies involving cancer cells [90]. Another study found that downregulation of miRNA-93 increases Nrf2 expression, reducing ROS and cell apoptosis and diabetic retinopathy [113]. Additionally, miRNA-153 promoted oxidative stress by inhibiting Nrf2 activity in an in vitro model of Parkinson´s disease [114]. As concerning Keap1, miR-141 was the first miRNA identified to suppress Keap1 levels in ovarian cancer cell lines [115]. Similarly, miR-432-3p was found to impair Keap1 mRNA translation in esophageal squamous cell carcinoma, positively modulating Nrf2 activity [116], while increased levels of miR-200a promoted Keap1 degradation and Nrf2 stabilization in OB-6 osteoblastic cells [109]. Overall, there is a growing number of studies supporting the potential use of miRNAs as an strategy to modulate the Nrf2/Keap1 pathway, either inhibiting (as it happens in cancer) or promoting the activation of Nrf2 as an antioxidant approach. Future research could benefit from these findings.

## 5. HO-1 Antioxidant Role in Atherosclerosis

HO-1 has many important roles that contribute to the protection against atherosclerosis, such as antioxidant, anti-inflammatory, antiapoptotic, or immunomodulatory effects [63]. HO-1 is expressed in the main cell types detected in human and mouse athe-rosclerotic lesions, including EC, macrophages, or SMCs [63,117].

HO-1 is a highly conserved enzyme—also known as 32-kDa heat shock protein—encoded by the hmox1 gene [18,118]. There are currently three isoforms of HO: HO-1, HO-2, and HO-3, although the last one seems to derive from HO-2 transcripts [119]. While HO-2 is constitutively expressed, HO-1 is usually present at low levels in many tissues but it can be stimulated by many factors [63]. For instance, HO-1 expression can be enhanced in response to ROS, ischemia-reperfusion, carcinogenesis, atherosclerosis itself, or other inflammatory processes [120]. Moreover, different transcriptor factors such as Nrf2, activator protein-1 (AP-1), or nuclear factor-kappa B (NFκB) have been shown to activate HO-1 expression, together with several up-stream kinases, like protein kinases A and C or phosphatidylinositol 3-kinase [120,121].

**Figure 4:**
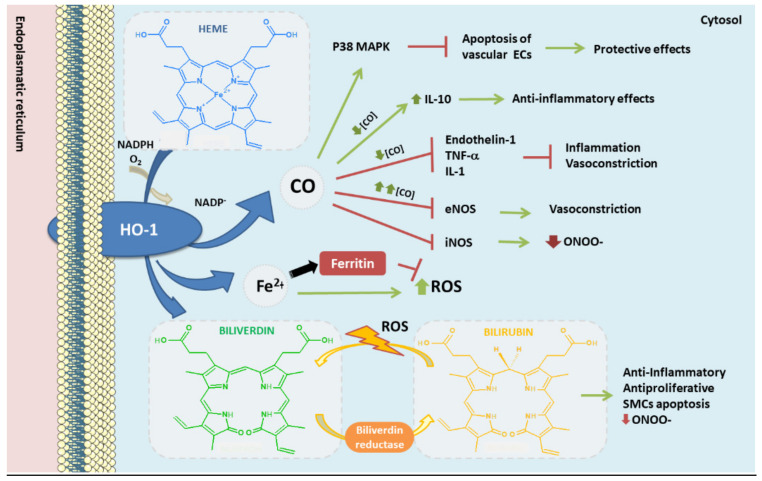
Heme oxygenase-1 antioxidant products derived from heme degradation. Heme oxyge-nase-1 (HO-1) catabolizes heme degradation into equimolar amounts of carbon monoxide (CO), Fe^2+^ and biliverdin. Biliverdin is converted to bilirubin by biliverdin reductase (BVR). All these products have shown antioxidant, anti-thrombotic and anti-inflammatory properties. In addition, HO-1 induces the production of ferritin, an iron storing protein—reducing the levels of Fe^2+^ which could derive into ROS via Fenton reaction.

In mammals, heme oxygenase enzymes are located on the surface of the endoplasmic reticulum, anchored to it by hydrophobic amino acids at its COOH-terminal ends. Its ca-talytic part contains a “heme binding pocket” of 24 amino acids, oriented to the cytosol [29,118], together with a histidine-imidazole residue where it binds to the heme iron [29]. HO-1 enzymatically catalyzes the oxidative cleavage of the heme group (mainly heme b) of metalloproteins in equimolar amounts of carbon monoxide (CO), Fe^2+^ and biliverdin IXα [29] (Figure 4). Biliverdin is then converted to bilirubin by the cytosolic biliverdin reductase (BVR). All these three products have anti-inflammatory, antioxidant, antiapoptotic, antithrombotic, and antiproliferative functions in vascular cells [63,122]. Thus, the anti-atherogenic role assigned to HO-1 appears to be associated, among others, with the bioproducts, biliverdin, bilirubin, and the vasodilator CO, providing arterial protection against oxidant-induced injury [122]. 

Different cell-based and in vivo research studies have demonstrated that HO-1 upregulation protects vascular walls from endothelial dysfunction and pathological remodeling, significantly inhibiting the atherosclerotic process [122,123,124,125,126]. Besides, diverse genetic population studies have indicated the importance of HO-1 expression as a protecting phenomenon against atherosclerosis [63]. Under basal conditions HO-1 is under-expressed in most tissues, however when a vascular injury occurs, the expression is higher [122]. High blood HO-1 levels have been found in different chronic diseases, such as diabetes mellitus [127] or Parkinson´s disease [14,15], mainly released into the plasma by different cell types in response to inflammation or oxidative stress [128]. Remarkably, the expression of HO-1 arising from macrophages during oxidative conditions was found higher in coronary artery disease (CAD) patients when compared to healthy subjects [129]. Likewise, HO-1 plasma levels are higher in patients with carotid plaques compared to those without plaques, and these levels increase with the severity of the plaques [130]. HO-1 expression is high in atherosclerotic lesions, correlating with plaque instability and pro-inflammatory markers [18]. However, such upregulation enhances the stabilization of atherosclerotic plaques, therefore representing an athero-protective mechanism [122,131]. Similarly, increased levels of bilirubin in plasma have been associated with lower incidence of CVDs [132] while low levels of bilirubin correlate with endothelial dysfunction and increased intima-media thickness [18,133], being inversely correlated the levels of bilirubin in serum with the severity of atherosclerosis in men [134]. Such anti--atherogenic role relies mainly on the antioxidant properties of bilirubin/biliverdin. Bilirubin seems capable to scavenge oxygen radicals and inhibit oxidation of LDL and other lipids, as well as presenting anti-inflammatory and anti-proliferative roles [63]. For instance, bilirubin enhances SMCs apoptosis, preventing its accumulation in vascular walls, and it blocks the recruitment and infiltration of leukocytes into these walls [135]. Finally, bilirubin cytoprotective action might be related to its capacity to inhibit inducible NOS (iNOS), reducing the production of the free radical ONOO^-^ [136]. 

In the same way, CO inhibits apoptosis in vascular ECs by activation of p38 mitogen-activated protein kinase (MAPK) signaling pathway [137]. Besides, CO blocks LPS-derived upregulation of iNOS and therefore an overproduction of NO in macrophages [138], modulating the response of these cells against bacterial LPS [139]. At low concentrations, CO inhibits vasoconstrictor enzymes, such as endothelin-1, as well as the production of TNFα and IL-1 (pro-inflammatory interleukin) and increases IL-10 (anti-inflammatory interleukin) [140]; while at high concentrations it has a vasoconstriction effect, inhibiting the action of eNOS. Among other properties of CO, the antioxidant role of this molecule might be due to its capability to bind Fe^2+^ in the heme group, preventing oxidation of hemoproteins, and avoiding the release of free heme [63,141].

The heme molecule itself is fundamental for many biological functions, due to its role as prosthetic group in hemoproteins like Hemoglobin (Hb), a major transporter of O_2_. However, free heme can be toxic for several reasons [142]. For instance, heme can affect vascular ECs integrity by oxidation of LDL [18,143], and it can enhance cellular cytotoxicity through different sources of ROS [144]. Moreover, free heme increases the chances of releasing Fe^2+^ from its porphyrin pocket [142], which can be oxidized to Fe^3+^ through the Fenton reaction, an important source of ROS. Fe^3+^ can promote LDL oxidation in presence of superoxide anion (O_2_^.₋^) [18]. Moreover, excessive amounts of iron can also modulate the activity of several enzymes directly involved in regulating cholesterol and triglyceride levels, which could have a negative effect over lipid metabolism [145]. For instance, different studies have demonstrated the correlation between iron overload and endothelial dysfunction, suggesting a critical role of redox active iron in the proinflammatory res-ponse of ECs [146]. Notably, elevated concentrations of iron can be found in atherosclerotic lesions compared to non-atherosclerotic vessels [29]. Therefore, the presence of redox active iron in atherosclerotic plaques may enhance the progression of atherosclerotic lesions by promoting an increase of free radicals, lipid peroxidation, and endothelial dysfunction.

Thus, the importance of heme degradation by HO-1 is notorious not only because it derives in the aforementioned antioxidant components (biliberdin/bilirubin, CO), it also reduces the levels of free heme molecules. HO-1 upregulation might represent a pro-oxidant process due to the degradation of heme and Fe^2+^ release. However, HO-1 stimulates the expression of ferritin, an iron storing protein (Figure 3). Indeed, ferritin colocalizes with HO-1 in atherosclerotic lesions, where they seem to play a protective antioxidant role [63,147]. Despite this, ferritin might exert pro-oxidant effects as well, as described [148].

Overall, HO-1 antioxidant activity can be explained by three key processes derived from its heme catalytic activity: production of biliverdin and related bilirubin, release of CO, and finally, stimulation of ferritin synthesis, therefore ensuring an optimal disposal/storage of Fe^2+^ derived from heme degradation. 

## 6. Therapeutic Approaches

Due to the beneficial properties linked to HO-1, different therapeutic approaches have been launched seeking HO-1 upregulation as a strategy to protect against oxidative stress and, for instance, to reduce vascular injury and atherosclerotic progression [135,149]. Indeed, several drugs like arginine and heme-arginate, or even gene therapy approaches, are currently applied to enhance HO-1 expression. To date, different studies have shown the potential of gene therapy targeting HO-1 overexpression as therapeutic approach. For example, the delivery of human HO-1 gene in SHR hypertensive rats resulted in long-term overexpression of this molecule and, moreover, attenuated hypertension in these animals [150]. Similarly, adenovirus-mediated expression of HO-1 within mesenchymal stem cells improved their survival and resistance to oxidative stress [151]. Moreover, HO-1 gene delivery has been proven effective to reduce vascular injury in animal models [152,153]. 

Alternatively, heme analogs and/or its derivatives also constitute effective inducers of HO-1 expression, mainly by binding and inactivating Bach1, which promotes Nrf2 nuclear translocation and specific HO-1 transcription [149,154]. For instance, administration of hemin prevents atherosclerotic lesion formation in LDL receptor knockout mice by inducing HO-1 [123,135]. Unfortunately, clinical trials using hemin have shown the short-term effect of this molecule, since the induction of HO-1 only lasts a few days [155,156]. Another inducer of HO-1 widely used is cobalt protoporphyrin. This molecule has contradictory effects, since it has proven to effectively induce HO-1 activity, but only transiently, while it can provoke adverse effects in several organs [149]. Besides, many “classical” drugs used in CVDs such as aspirin, statins or paclitaxel, appear to induce HO-1 gene expression [135], although the variety of results found within clinical trials suggest that further research is required in this field [149]. Finally, diverse natural antioxidants—such as curcumin, quercentin, or lipoic acid—have been found to induce HO-1 expression [121]. Curcumin (diferuloylmethane), a polyphenol present in the root of *Curcuma (Curcuma longa*), has been shown to induce HO-1 expression by activation of the Nrf2/ARE pathway in a dose- and time-dependent manner [157]. Besides, curcumin might also promote HO-1 upregulation through PI3K/Akt signaling [158]. Unfortunately, several studies have reported mutagenic effects (mtDNA and nuclear DNA damage) when using high doses of curcumin. Moreover, the low stability and poor absorption of this molecule has derived in the design of synthetic analogs instead. Similarly, the polyphenol resveratrol (3,5,4′-trihydroxystilbene) also induces HO-1 expression via inhibition of PI3K/Akt pathway [159] or activation of Nrf2 [160]. Remarkably, resveratrol attenuated inflammation and oxidative stress in a rat model of myocardial ischemia/reperfusion (I/R) injury by activating Nrf2 and enhancing HO-1 expression [161]. Although the low bioavailability of resverastrol has limited its use [162], several analogs of this compound, such as piceatannol, have also been shown to induce HO-1 expression [163]. In addition, a derivative of resveratrol, trans-3,5,4´-trimethoxystilbene, has shown atheroprotective effects, by suppressing cholesterol accumulation in macrophage foam cells [164]. Similarly, epigalloca-techin gallate (EGCG)—a polyphenol found in green tea that also enhances Nrf2/HO-1 pathway—might ameliorate the development of atherosclerosis by reducing ECs apoptosis and ROS production as well as inhibit inflammation [165]. Finally, activation of the Nrf2 pathway by the natural oil Z-Ligustilide (Z-Lig) appears to attenuate oxidative stress in human keratinocytes [166] or alleviating cerebral ischemia [167]. Moreover, by activation of the Nfr2/ARE signaling pathway, Z-Lyg reduced oxidative stress in EA-hy926 cells and also ameliorated endothelial dysfunction and atherosclerotic plaque formation in HFD-fed Ldlr null mice [168]. Overall, these and other natural compounds have proven their potential to reduce ROS production by modulation of the HO-1/Nrf2 system or si-milar pathways, although further studies are required in order to confirm the safety and also to implement the efficacy of these approaches [169]. 

Direct administration of biliverdin/bilirubin or CO and CO releasing molecules (CORMs) are other strategies explored to treat vascular injury [135]. Indeed, several preclinical studies have shown the beneficial effects of CO inhalation (iCO). For example, a phase I dose trial with iCO demonstrated the safety of low-dose iCO administration in patients with sepsis induced acute respiratory distress syndrome [170], were HO-1 might play a protective role [171]. Furthermore, this trial also indicated that the circulating levels of mtDNA were lower in the iCO treated groups compared to untreated controls [170]. Derived from it, a phase II clinical trial is currently recruiting (NCT03799874, www.clinicaltrials.gov, accessed on 25 August 2021). Alternative strategies for the delivery of this compound such as membrane-based controlled systems to release CO are being tested [172], together with the application of CORMs, which allow to release CO in a controlled manner [173]. 

Considering that the modulation of HO-1 expression is linked to Nrf2, pharmacological drugs are also applied to directly activate Nrf2/HO-1 signaling pathway instead [149]. It this sense, activation of Nrf2 by canagliflozin (a sodium-glucose co-transporter-2 inhibitor) enhances HO-1 expression, together with anti-proliferative and anti-migratory effects on SMCs [174]. Other molecules, such as the hormone melatonin, have also been shown to enhance the expression of Nrf2. For instance, a randomized controlled trial indicated that melatonin could significantly ameliorate brain I/R injury by enhancing the expression of antioxidant proteins such as Nrf2 in patients after carotid endarterectomy (NCT03115034, www.clinicaltrials.gov, accessed on 25 August 2021) [175]. In addition, many electrophilic molecules that react with the cysteine thiols of Keap1, are used as Nrf2 inducers. Such is the case of fumaric acids such as dimethyl fumarate and monomethyl fumarate, the most prominent Keap1 residue modifiers [176]. Dimethyl fumarate has been tested in several clinical trials for cutaneous T cell lymphoma (NCT02546440, phase II), multiple sclerosis (NCT02461069, phase IV), or chronic lymphocytic leukemia (NCT02784834, phase I), among others. Similarly, bardoxolone methyl, a synthetic triterpenoid that activates Nrf2, has been tested in patients with diabetic nephropathy (NCT00664027, phase II), pulmonary hypertension (NCT03068130, phase III) or in chronic kidney disease/type 2 diabetes (NCT00811889, phase II). Finally, sulforaphane, an isothiocyanate present in broccoli, has shown to induce Nrf2-HO-1/NQO-1 signaling pathway [177], and its effect has been eva-luated in several diseases, such as Type 2 diabetes (NCT02801448, phase II) or metastatic breast cancer (NCT02970682, phase II). 

Of note, any pharmacological strategy targeting HO-1 should take into account that Nrf2 activation could also derive in undesired pro-atherogenic effects [63], and moreover, Nrf2 activators might also accelerate tumor progression and metastasis [72] or other undesirable effects. Thus, although many studies have proven the efficacy of these therapies to tackle vascular disease, mainly in animal models, further research is required in this field, since many factors and concerns need to be addressed still before any successful translation to the clinic. 

## 7. Conclusions

Oxidative stress represents a key player in the pathophysiology of atherosclerosis. The imbalance between pro- and antioxidant agents drives to an overproduction of ROS, which promotes DNA and lipid peroxidation, cell death, and endothelial dysfunction, initial steps in the development of atherosclerotic plaques. In order to reduce ROS production and tackle oxidative stress and atherosclerosis itself, many researchers have focused on identifying mechanisms to activate the Nrf2/HO-1 signaling pathway. Overall, both, Nrf2 and HO-1 have shown to protect against atherosclerotic injury through their antioxidant, anti-inflammatory, or even immunomodulatory properties. Different strategies based either in the modulation of Nrf2 by its interaction with Keap 1 or alternatively through other pathways involving Nrf2 phosphorylation or epigenetic modulation via miRNAs are currently under research. In addition, many natural compounds like resveratrol or sulforaphane are also being tested due to their potential to activate Nrf2/HO-1 pathway. Some of these molecules have shown promising results at the preclinical side. On the other hand, Nrf2 may also enhance atherosclerosis through additional mechanisms that need to be further characterized. Thus, further research in this field is required, in order to better understand the involvement of Nrf2/HO-1 in the pathophysiology of atherosclerosis and moreover, to identify therapeutic alternatives to trigger their antioxidant and anti-atherogenic effects.

## Figures and Tables

**Table 1:** Modulators of Nrf2. Protein kinases and miRNA known to regulate Nrf2 activity are shown. Acronyms: GSK-3β: Glycogen synthase kinase-3β; FYN: Proto-oncogene tyrosine-protein kinase Fyn; p38: p38 MAP kinase; PI3K/Akt: Phopshoinositide 3-kinase/Protein kinase B (Akt); PKC: Protein kinase C; JNK: c-Jun NH2-terminal protein kinase; ERK: Extracellular signal-regulated kinase; PERK: Protein kinase R (PKR)-like endoplasmic reticulum kinase; AMPK: AMP-activated protein kinase; miRNA: micro RNA.

Protein Kinases	Identified in	Effect	References
GSK-3β	HEK293T	Inhibits NRF2	[93] Rada et al., 2011 [97] Salazar et al., 2006
	HepG2	Inhibits NRF2	[98] Jain et al., 2006
FYN	HepG2	Inhibits NRF2, via GSK-3B	[92] Jain et al., 2007
P38 MAP kinase	Thymocytes and 293T; HepG2	Inhibits HO-1/Nrf2 Activates HO-1/Nrf2	[99] Thornton et al., 2008; [100] Shen et al., 2004 [101] Elbirt et al. 1998
PI3k /Akt	293T	Activates NRF2, by Gsk-3β inhibition.	[97] Salazar M et al. 2006
PKC	HepG2	Activates NRF2	[95] Huang et al., 2002
JNK	HepG2	Activates NRF2	[100] Shen et al., 2004
ERK	HepG2	Activates NRF2, through GSK-3β inhibition.	[100] Shen et al., 2004 [101] Elbirt et al., 1998
PERK	Mouse embryonic fibroblasts	Activates NRF2	[102] Cullinan et al. 2003
AMPK	HepG2, HEK293	Activates Nrf2	[96] Joo et al. 2016
miRNA	Identified in	Effect	**References**
miR-28	Breast cancer cell lines	Inhibits	[103] Yang et al., 2011
miR-34a	Hepatocytes	Inhibits	[104] Huang et al., 2014
	Cardiomyocytes		[105] Wang et al., 2019
miR-132, miR-200c	Renal proximal tubular cell line	Inhibits	[106] Stachurska et al., 2012
miR-144	Neuronal cell lines; Lymphoblast cell lines (k562 cell line)	Inhibits	[107] Zhou et al., 2017
miR-153, miR27a, miR-142-5p	Neuronal cell lines	Inhibits	[108] Narashimhan et al., 2012
miR-200a	OB-6 osteoblastic cells	Increases	[109] Zhao et al., 2017
miR-140-5p	HK2 cells	Increases NRF2 expression	[110] Liao W et al., 2017
miR873-5p	Mouse renal tubular epithelial cells (mRTECs)	Increases NRF2 and HO-1 expression	[111] Wang J et al., 2019

## Data Availability

Not applicable.
